# Impaired metabolic effects of metformin in men with early-onset androgenic alopecia

**DOI:** 10.1007/s43440-021-00347-8

**Published:** 2021-12-12

**Authors:** Robert Krysiak, Karolina Kowalcze, Bogusław Okopień

**Affiliations:** 1 Department of Internal Medicine and Clinical Pharmacology, School of Medicine in Katowice, Medical University of Silesia, Medyków 18, 40-752 Katowice, Poland; 2 Department of Pediatrics in Bytom, School of Health Sciences in Katowice, Medical University of Silesia, Katowice, Poland

**Keywords:** Androgen excess, Insulin sensitivity, Men’s health, Metformin, Prediabetes, Risk factors

## Abstract

**Background:**

Early-onset androgenic alopecia is considered the phenotypic equivalent of polycystic ovary syndrome in men. The purpose of the current study was to investigate whether the presence of early-onset male-pattern baldness modulates metabolic effects of metformin.

**Methods:**

This prospective case–control study included 2 groups of men at high risk for type 2 diabetes: 72 individuals with androgenic alopecia (group A) and 75 subjects with normal hair growth (group B). Both groups were matched for age, blood pressure, body mass index, insulin sensitivity and plasma lipids. Glycated hemoglobin, glucose, plasma lipids, indices of insulin sensitivity/resistance, sex hormones, high-sensitivity C-reactive protein (hsCRP) and 25-hydroxyvitamin D were determined before and after metformin treatment (1.7 g daily).

**Results:**

Twelve-month metformin treatment reduced fat content, waist circumference, glycated hemoglobin, glucose and triglycerides, as well as improved insulin sensitivity. Although observed in both study populations, these effects were more pronounced in group B. Moreover, metformin decreased hsCRP and bioavailable testosterone levels in group B, as well as reduced 25-hydroxyvitamin D concentration in group A. Treatment-induced changes in glucose homeostasis markers correlated with the impact of metformin on hsCRP and 25-hydroxyvitamin D levels.

**Conclusions:**

Metabolic effects of metformin in males are attenuated if they have coexisting early-onset androgenic alopecia. This finding may be partially explained by differences in severity of low-grade systemic inflammation and vitamin D status. The obtained results, requiring confirmation in large prospective studies, suggest that men with early-onset male-pattern baldness benefit to a lesser degree from metformin treatment than other men at high risk for type 2 diabetes.

**Supplementary Information:**

The online version contains supplementary material available at 10.1007/s43440-021-00347-8.

## Introduction

Androgenic hair loss is the most common form of baldness in Caucasian men, affecting up to 30% of individuals by the age of 30 and 80% of men in the course of their life [1, 2]. Male-pattern hair loss diagnosed before the age of 30–35 years and reaching at least stage 3 of Hamilton-Norwood classification is termed as early-onset androgenic alopecia [3]. This disorder is a frequent finding in first-generation male relatives of women with polycystic ovary syndrome [4], and both disorders share similar genetic backgrounds (CAG and GGC triplet repetition polymorphisms, *WNT10A* gene mutations and *APCDD1* gene mutations) [5, 6]. Moreover, hormonal and metabolic abnormalities characterizing early-onset androgenic alopecia (elevated levels of testosterone, dehydroepiandrosterone-sulfate [DHEA-S], luteinizing hormone [LH] and prolactin, decreased levels of follicle-stimulating hormone [FSH] and sex hormone-binding globulin, higher values of the free androgen index and the LH/FSH ratio, and insulin resistance) resemble those observed in women with polycystic ovary syndrome and their brothers [7–9]. Because of these similarities, male-pattern hair loss is regarded as the male equivalent of polycystic ovary syndrome [10, 11].

Androgenic alopecia is an independent predictor of mortality for type 2 diabetes and cardiovascular diseases [12], while its severity correlates with the risk of coronary artery disease [13]. The mechanism of this increase has not been identified but may be associated with metabolic syndrome, frequently coexisting with early-onset male-pattern hair loss [14–16]. This syndrome, linked to insulin resistance, is a specific cluster of risk factors for cardiovascular and metabolic disorders [17]. Compared with the general population, subjects with early-onset androgenic alopecia are more frequently diagnosed with all components of metabolic syndrome: obesity, overweight, insulin resistance, dyslipidemia and hypertension [10, 14]. Impaired insulin sensitivity was observed even after exclusion from the analysis of obese individuals [9]. Moreover, subjects with male-pattern hair loss were more frequently treated with anti-hypertensive agents than men with normal hair growth [18]. The mechanisms underlying the association between androgenic alopecia and metabolic syndrome have not been fully understood but seem complex. Increased testosterone production, enhanced conversion of testosterone to dihydrotestosterone and increased transcriptional activity of the androgen receptor result in both shortening of the anagen phase of hair growth, as well as in impairment of glucose uptake and incorporation into glycogen in skeletal muscles, associated with insulin resistance [2]. In turn, insulin resistance, decreasing hepatic sex hormone-binding globulin production, increases free androgen levels, leading to follicular miniaturization and alopecia [19]. It is also possible that both increased androgen levels and insulin resistance are independently related to low-grade systemic inflammation [20, 21], while alopecia is only an epiphenomenon reflecting androgen excess and/or insulin resistance.

Recently, Krysiak et al. [22] have reported that rosuvastatin, a 3-hydroxy-3-methyl-glutaryl-CoA **r**eductase inhibitor, strongly lowering total and LDL cholesterol levels and highly effective in the primary and secondary prevention of coronary artery disease and stroke [23], deteriorated insulin sensitivity and induced less pronounced cardiometabolic effects in men with early-onset male-pattern hair loss than in subjects with normal hair growth. Similarly, both hypotensive and pleiotropic effects of lisinopril, one of the most commonly used angiotensin-converting enzyme inhibitors [24], in hypertensive individuals were less pronounced if they had coexisting early-onset androgenic alopecia [25]. Metformin is the first-line therapeutic agent for type 2 diabetes mellitus, as well as a drug commonly used in metabolic syndrome, prediabetes and polycystic ovary syndrome [26]. However, its glucose-lowering effect is highly variable and as many as 20–50% of patients initiated on metformin treatment never achieve glycemic goals [27]. Therefore, the aim of the current study was to investigate whether its presence modulates metabolic effects of metformin in men with impaired glucose tolerance (IGT) who are at high risk for diabetes. The primary study objective was to compare the effect of metformin treatment on glycated hemoglobin between men with early-onset male-pattern baldness and men with no evidence of hair loss. In addition, the effects of metformin on the remaining markers of glucose homeostasis, plasma lipids, hormones, low-grade systemic inflammation, vitamin D status, anthropometric measures and blood pressure were compared. The choice of treatment duration resulted from our previous observations indicating that metformin administered at the dose used in the current study, exerts its maximum effect on glucose homeostasis after 12 months of treatment, remaining later at a similar level (Krysiak et al., unpublished data). The same treatment period was also chosen by other research groups in studies including individuals with IGT [28, 29].

## Materials and methods

The research was conducted in accordance with the Declaration of Helsinki and was approved by the local review board (the Bioethical Committee of the Medical University of Silesia [KNW/0022/KB/234/17]; October 17, 2017). All participants provided written informed consent after the investigator had explained the nature, significance and implications of the study, as well as had informed patients about the potential benefits and risks associated with participation in the study. Owing to the non-randomized nature of the study, as well as because the assignment of the medical intervention was not at the discretion of the investigators, the study did not meet the criteria of a clinical trial and did not require registration prior to patient enrollment.

### Study population

**Fig. 1:**
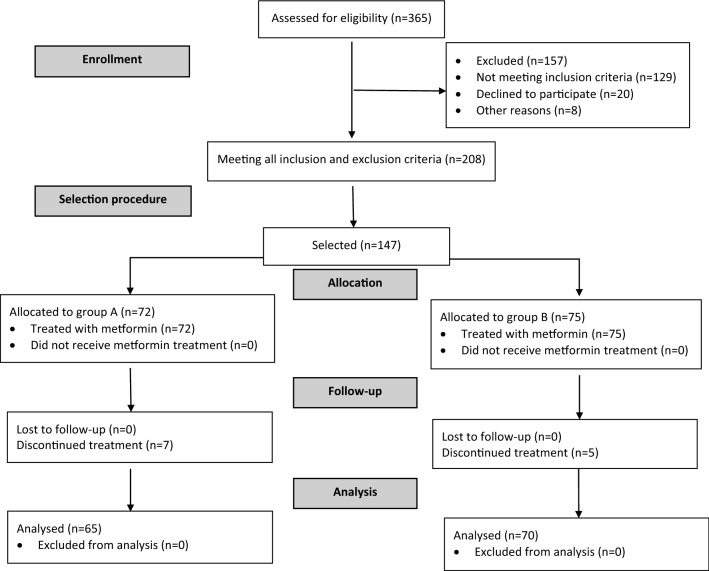
A diagram presenting the flow of patients in the study

The participants of this prospective case–control study were recruited among 365 men (25–50 years old) with: (a) body mass index (BMI) at least 24 kg/m^2^, (b) plasma glucose concentration 2 h after 75-g glucose challenge in the range between 140 and 200 mg/dL, and (c) fasting plasma glucose levels in the range between 95 and 125 mg/dL. These inclusion criteria were similar to those used in the landmark Diabetes Prevention Program trial [30]. The potential participants, initially supervised by local healthcare providers cooperating with our research team, were considered eligible for enrollment if they had been diagnosed with IGT 6–12 months prior to commencement of the study, had complied for at least 6 months with the lifestyle modification, and had not been treated with any hypoglycemic agents in the past.^1^ The study participants (*n* = 147) were assigned to one of two groups. The sample-size calculation showed that at least 60 men in each group were necessary to detect a 20% between-group difference in the primary endpoint with 90% power and a significance level of 5%. To compensate possible drop-outs or losses to follow-up, the sample size was increased by 20–25%. Group A included 72 individuals with early-onset male-pattern baldness, defined as grade 3 vertex or more alopecia diagnosed before the age of 30 years. Group B included 75 men with no evidence of hair loss and served as a control group. The hair status was classified using the Norwood modification [31] of the Hamilton Classification Scale for androgenic alopecia [32]. The participants were selected among 208 men considered eligible for enrollment by means of a computer algorithm to create 2 groups matched for age, BMI, blood pressure, plasma lipid levels and glucose homeostasis markers. To minimize the effect of seasonal fluctuations in outcome measures, recruitment took place over a 1-year period. The flow of patients through the study is depicted in Fig. 1.

The exclusion criteria were as follows: diabetes, thyroid disorders or other endocrine disorders; kidney or liver insufficiency; chronic inflammatory or autoimmune disorders; malabsorption syndromes; cardiovascular disease (with the exception of mild arterial hypertension) and other serious disorders. We also excluded individuals treated within 6 months preceding the study with any prescription or over-the-counter drug for more than a week. Diabetes was an exclusion criterium because its presence often results in chronic complications (nephropathy, retinopathy, neuropathy and early atherosclerosis) that may affect metformin action; metformin dose in diabetes varies from patient to patient (depending on disease severity) and is often titrated based on the patient’s clinical response and tolerability; and because subjects with diabetes are often treated with many different drugs (other anti-diabetic agents, acetylsalicylic acid, statins, angiotensin-converting enzyme inhibitors, sartans and/or β-blockers) that may affect metabolic effects of metformin. Other reasons for exclusion were as follows: thyroid disorders or other endocrine disorders may affect insulin sensitivity, may alter sex hormone levels, and/or often require supplemental therapy, potentially affecting metformin action; kidney failure, liver failure and other serious disorders are contraindications for metformin use; chronic inflammatory or autoimmune disorders may modify insulin sensitivity by inducing chronic inflammation; malabsorption syndromes impair metformin absorption and are often associated with poor metformin tolerance; cardiovascular disease (with the exception of mild arterial hypertension) either directly or indirectly (by drug interactions) affects the pharmacokinetics of metformin; while chronically used prescription or over-the-counter drugs may exert metabolic effects or interact with metformin.

### Study design

Treatment with metformin was initiated at a dose of 850 mg taken orally once daily. After 4 weeks, the dose of metformin was increased to 850 mg twice daily and no changes in dosage were allowed for the following 11 months of the study. All included patients were informed that metformin must be swallowed whole and not crushed and chewed. To minimize potential adverse effects of metformin, the drug was swallowed with or immediately after meals. Short-term use of any prescription or over-the-counter drugs (not longer than 7 days) was accepted only if such treatment was terminated at least 6 weeks before the end of the study. All participants were given detailed advice about how to achieve the goals of lifestyle modification, which were a reduction in weight of 7% or more if necessary; total fat intake less than 30% of total energy intake; saturated fat intake less than 7% of energy consumed; cholesterol intake less than 200 mg per day; and an increase in fiber intake to 15 g per 1000 kcal. They were also requested to do at least 150 min of moderate-intensity aerobic physical activity per week, and to do muscle-strengthening activities that were moderate intensity and involved all major muscle groups on 2 or more days a week. Participants were seen every 8 weeks to ensure adherence to metformin treatment and to boost compliance with the study protocol. They were considered adherent to their medication if the number of tablets returned during each visit divided by the number of pills prescribed by the investigator was in the range from 0 to 0.1 and all questions in the Morisky–Green test were correctly answered. Compliance with non-pharmacological recommendations was measured by analysis of individual dietary questionnaires and of diaries in which the patients recorded all their activities.

### Anthropometric and blood pressure measurements

BMI was calculated as weight (in kilograms) divided by height (in meters) squared. Fat-free mass was measured using bioelectrical impedance analysis (model BF-511 B**,** Omron Healthcare Europe**,** Hoofddorp, the Netherlands), which is based on different conductance and impedance of fat and fat-free tissue. Fat-free mass index was calculated using the following equation: fat free mass/height squared**.** The waist circumference was measured at the end of a normal expiration midway between the lower margin of the last palpable rib and the top of the iliac crest, using a stretch‐resistant tape, and rounded to the nearest 1 cm. During measurements, the tape, providing constant tension through the use of a special indicator buckle, was snug (but did not compress the skin) and was parallel to the floor. Blood pressure was measured on the nondominant arm at 5-min intervals and the values used in statistical analyses were the means of three measurements. The use of the non-dominant arm minimized the impact of asymmetric arm development and function (a greater muscle volume and activity in the dominant arm), giving a more accurate blood pressure reading by a sphygmomanometer.

### Laboratory assays

All laboratory assays were carried out at the beginning of the study and 12 months later. To minimize the risk of detection bias, all measurements were carried out in duplicate by persons blinded to patients' clinical status and the study protocol. Venous blood samples were collected between 7.00 and 8.30 a.m. after 12-h overnight fasting in a quiet and air-conditioned room (constant temperature of 23–24 °C). Before venipuncture, all individuals had been resting in the seated position for at least 30 min. Glucose and insulin levels were additionally determined in samples collected 60 and 120 min after consumption of 75 g of glucose. Plasma levels of glucose, creatinine, total cholesterol, low-density lipoprotein (LDL) cholesterol, high-density lipoprotein (HDL) cholesterol, triglycerides and albumin were measured by routine laboratory techniques using commercially available kits (Roche Diagnostics, Basel, Switzerland). Plasma concentrations of insulin, androgens (total testosterone and DHEA-S), estradiol, sex-hormone binding globulin and 25-hydroxyvitamin D were assayed using an electrochemiluminescent analyzer (Cobas e411, Roche Diagnostics, Mannheim, Germany). Plasma levels of high-sensitivity C-reactive protein (hsCRP) were measured by immunoassay with chemiluminescent detection (Immulite 2000XPi, Siemens Healthcare, Warsaw, Poland). Glycated hemoglobin was measured in whole blood samples using turbidimetric inhibition immunoassay on the Cobas Integra 800 analyzer (Roche Diagnostics, Mannheim, Germany). Bioavailable testosterone was calculated from total testosterone and sex hormone-binding globulin levels with the Vermeulen formula, by means of the online calculator available at www.issam.ch/freetesto.htm. All indices of insulin sensitivity/resistance and β-cell function: the homeostatic model assessment 1 of insulin resistance (HOMA)1-IR, HOMA2-IR, HOMA2-%β, HOMA2-%S, the quantitative insulin sensitivity check index (QUICKI), Matsuda index and Stumvoll (0–120) index were calculated as described elsewhere [33, 34]. The estimated glomerular filtration rate was calculated from creatinine levels using the abbreviated Modification of Diet in Renal Disease study equation.

### Statistical analysis

The outcome variables were log-transformed to achieve a normal distribution prior to statistical analysis. Between‐group comparisons in each time point, as well as comparisons of percent changes from baseline after adjustment for baseline values were performed using Student's *t* tests for independent samples, while within‐group comparisons were made using Student's paired *t*-test. Categorical data were compared using the *χ*^2^ test. The strength of relationships between the measured variables was estimated using Pearson's *r*-tests. *P* values corrected for multiple testing below 0.05 were considered statistically significant.

## Results

At the beginning of the study, there was no difference between the study arms in terms of age, smoking, duration of IGT, BMI, fat-free mass index, fat content, waist circumference and blood pressure. The two groups were comparable with regard to glycated hemoglobin, fasting and 2-h post-load plasma glucose, insulin sensitivity, plasma lipids, estradiol and the glomerular filtration rate. Total testosterone, bioavailable testosterone, DHEA-S and hsCRP levels were higher in group A than in group B, while the opposite relationship was observed for 25-hydroxyvitamin D levels (Table 1).

**Table 1:** Baseline characteristics of patients

Variable	Group A^a^	Group B^b^	*p* value
Number [*n*]	65	70	–
Age [years; mean (SD)]	37 (6)	38 (6)	
Smokers [%]	35	34	–
Duration of IGT [moths; mean (SD)]	9 (2)	9 (2)	1.0000
BMI [kg/m^2^; mean (SD)]	32.9 (5.3)	32.4 (5.1)	0.5774
Fat-free mass index [kg/m^2^; mean (SD)]	19.0 (2.0)	18.9 (1.9)	0.7662
Fat content [%; mean (SD)]	42.2 (4.9)	41.6 (4.3)	0.4501
Waist circumference [cm; mean (SD)]	108 (8)	107 (7)	0.4401
Systolic blood pressure [mmHg; mean (SD)]	125 (11)	124 (10)	0.5810
Diastolic blood pressure [mmHg; mean (SD)]	80 (7)	79 (7)	0.4082
Glycated hemoglobin [%, mean (SD)]	6.10 (0.18)	6.12 (0.15)	0.4832
Fasting glucose [mmol/L; mean (SD)]	6.16 (0.41)	6.11 (0.38)	0.4634
2-h post-load glucose [mmol/L; mean (SD)]	9.56 (0.78)	9.44 (0.84)	0.3924
HOMA1-IR [mean (SD)]	4.38 (1.21)	4.26 (1.25)	0.5724
HOMA2-IR [mean (SD)]	2.16 (0.32)	2.11 (0.29)	0.3427
HOMA2-%β [mean (SD)]	46.6 (7.1)	47.4 (7.5)	0.5263
QUICKI [mean (SD)]	0.308 (0.024)	0.309 (0.030)	0.8318
Matsuda index [mean (SD)]	1.87 (0.32)	1.91 (0.29)	0.4475
Stumvoll index [mean (SD)]	0.0257 (0.0058)	0.0273 (0.0062)	0.1246
Total cholesterol [mmol/L; mean (SD)]	5.25 (1.32)	5.12 (1.21)	0.5515
HDL cholesterol [mmol/L; mean (SD)]	1.15 (0.24)	1.18 (0.23)	0.4597
LDL cholesterol [mmol/L; mean (SD)]	3.20 (0.70)	3.12 (0.60)	0.4762
Triglycerides [mmol/L; mean (SD)]	1.91 (0.71)	1.81 (0.56)	0.3634
Total testosterone [nmol/L; mean (SD)]	23.8 (6.0)	17.0 (3.8)	** < 0.0001**
Calculated bioavailable testosterone [nmol/L; mean (SD)]	11.3 (3.5)	7.5 (2.0)	** < 0.0001**
DHEA-S [nmol/mL; mean (SD)]	7.0 (2.2)	5.2 (1.5)	** < 0.0001**
Estradiol [pmol/L; mean (SD)]	150 (38)	148 (46)	0.7843
hsCRP [nmol/L; mean (SD)]	34.2 (10.8)	29.8 (8.8)	**0.0103**
25-hydroxyvitamin D [nmol/L; mean (SD)]	59.0 (12.8)	69.4 (14.5)	** < 0.0001**
Estimated glomerular filtration rate [ml/min/1.73 m^2^; mean (SD)]	110 (20)	112 (21)	0.5725

Only data of 135 individuals who completed the study were included in the final analyses. Although all values were natural-log transformed, the table shows the raw data because the mean and SD values of log-transformed data are less relevant. Both groups were compared using Student’s t-test for independent samples (quantitative data) or using the *χ*^2^ test (categorical data)

Statistically significant results are marked in bold

*P* values corrected for multiple testing below 0.05 were considered statistically significant

^a^Men with early-onset androgenic alopecia

^b^Men with normal hair growth

Four patients (three in group A and one in group B) developed diabetes and required treatment modification. A further three patients (all from group A) were unable to complete the study period because of troublesome adverse effects associated with metformin therapy (abdominal bloating, vomiting, diarrhea and dysgeusia). One man with alopecia and three with normal hair growth dropped out during the follow-up because of non-compliance with the study protocol. Finally, one subjects (assigned to group B) withdrew consent because of personal reasons. Neither significant adverse effects nor any other complications were reported for the remaining 135 patients (92%) who completed the study protocol and were included in the final analyses. Over the entire study period, all these patients complied with dietary recommendations and recommendations concerning the required physical activity. The analysis of diaries showed that the average daily intake of calories, total fat, saturated fat and cholesterol intake, as well as physical activity did not differ between the study groups.

**Fig. 2:**
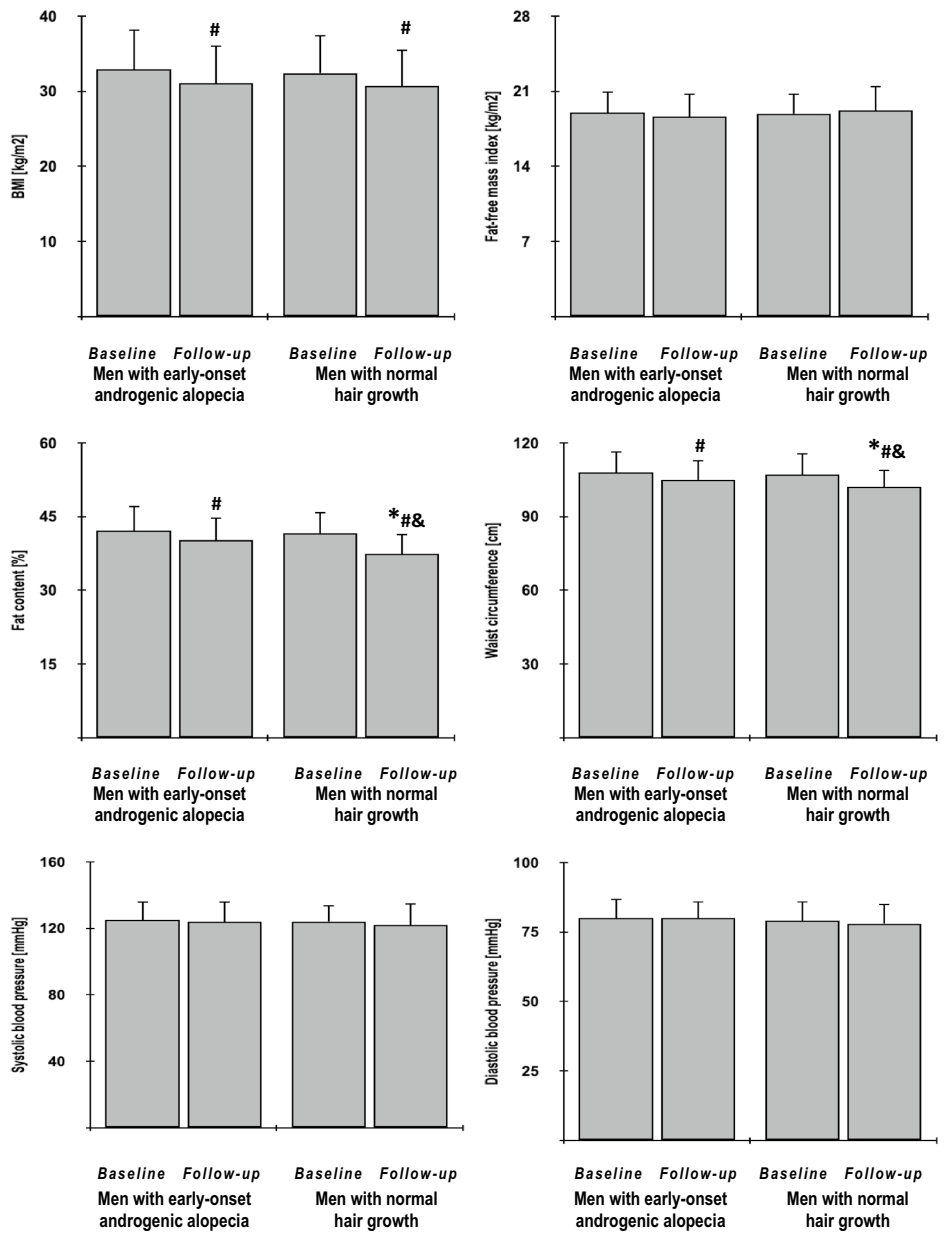
The impact of metformin on anthropometric measures and blood pressure in the study population. Only data of 135 individuals who completed the study: 65 men with early-onset androgenic alopecia (group A) and 70 men with normal hair growth (group B) were included in the final analyses. Although all values were natural-log transformed, the figure shows the raw data because the mean and SD values of log-transformed data are less relevant. Both groups were compared using Student’s *t*-test for independent samples. Differences between post-treatment (follow-up) and baseline values in each treatment group were identified using Student’s paired t-test. Comparisons of percent changes from baseline after adjustment for baseline values (reflecting the strength of metformin action) were performed using Student's *t* tests for independent samples. *p* values corrected for multiple testing below 0.05 were considered statistically significant. *p < 0.05 vs. men with early-onset androgenic alopecia in the same time point (group A); ^#^*p* < 0.05 vs. baseline value in the same treatment group; ^&^*p* < 0.05 vs. percent changes from baseline after adjustment for baseline values in group A

**Fig. 3:**
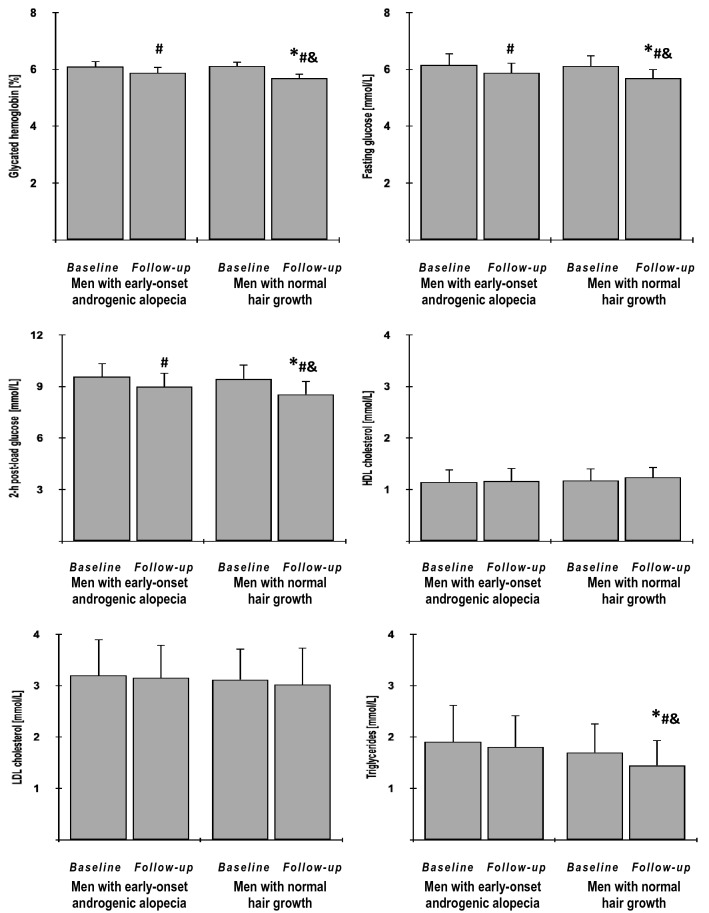
The impact of metformin on glycated hemoglobin, fasting and post-load glucose and plasma lipids in the study population. Only data of 135 individuals who completed the study: 65 men with early-onset androgenic alopecia (group A) and 70 men with normal hair growth (group B) were included in the final analyses. Although all values were natural-log transformed, the figure shows the raw data because the mean and SD values of log-transformed data are less relevant. Both groups were compared using Student’s t-test for independent samples. Differences between post-treatment (follow-up) and baseline values in each treatment group were identified using Student’s paired t-test. Comparisons of percent changes from baseline after adjustment for baseline values (reflecting the strength of metformin action) were performed using Student's t tests for independent samples. *p* values corrected for multiple testing below 0.05 were considered statistically significant. *p < 0.05 vs. men with early-onset androgenic alopecia in the same time point (group A); ^#^*p* < 0.05 vs. baseline value in the same treatment group; ^&^*p* < 0.05 vs. percent changes from baseline after adjustment for baseline values in group A

**Fig. 4:**
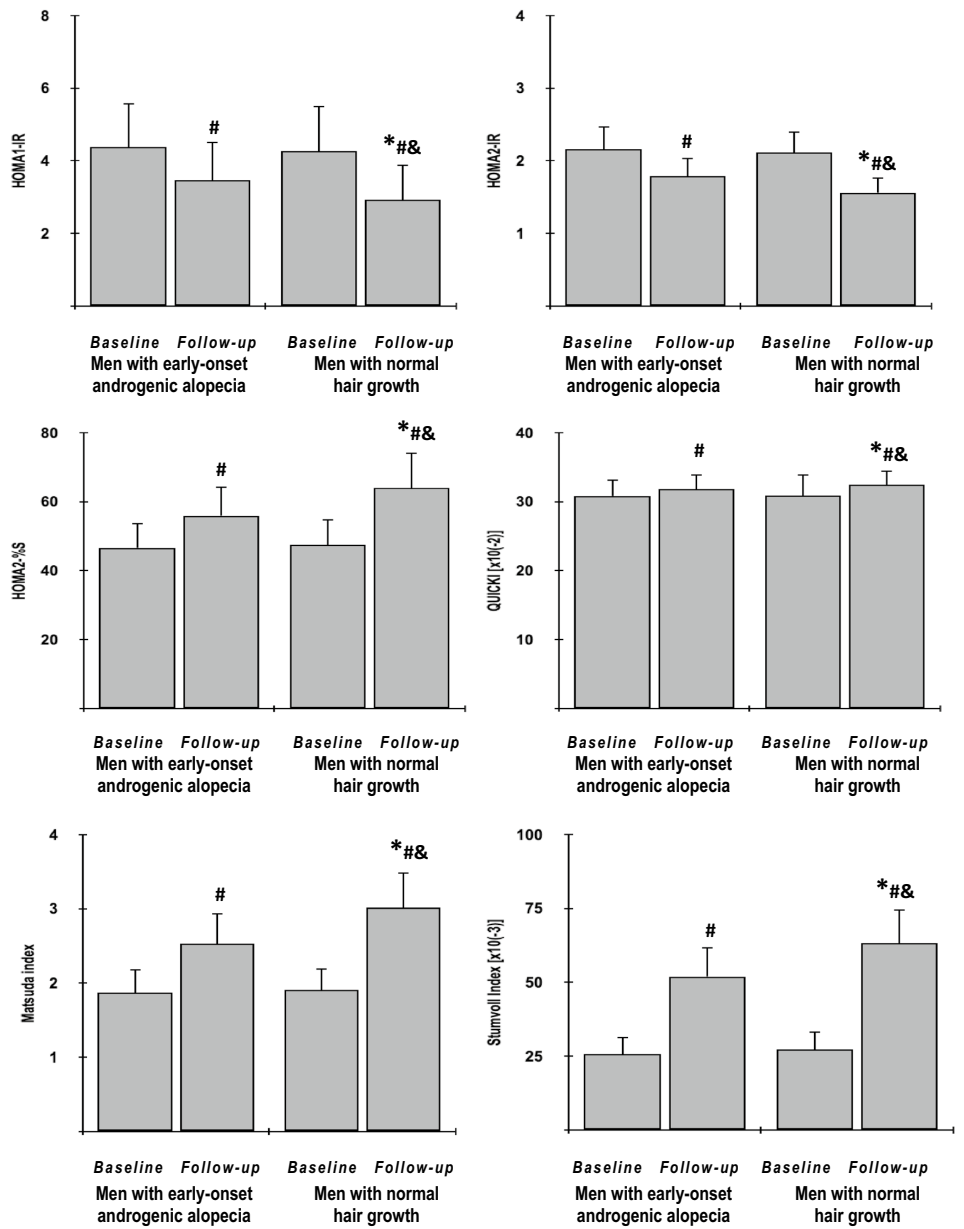
The impact of metformin on indices of insulin sensitivity/resistance in the study population**.** Only data of 135 individuals who completed the study: 65 men with early-onset androgenic alopecia (group A) and 70 men with normal hair growth (group B) were included in the final analyses. Although all values were natural-log transformed, the figure shows the raw data because the mean and SD values of log-transformed data are less relevant. Both groups were compared using Student’s t-test for independent samples. Differences between post-treatment (follow-up) and baseline values in each treatment group were identified using Student’s paired *t*-test. Comparisons of percent changes from baseline after adjustment for baseline values (reflecting the strength of metformin action) were performed using Student's t tests for independent samples. *p* values corrected for multiple testing below 0.05 were considered statistically significant. *p < 0.05 vs. men with early-onset androgenic alopecia in the same time point (group A); ^#^*p* < 0.05 vs. baseline value in the same treatment group; ^&^*p* < 0.05 vs. percent changes from baseline after adjustment for baseline values in group A

**Fig. 5:**
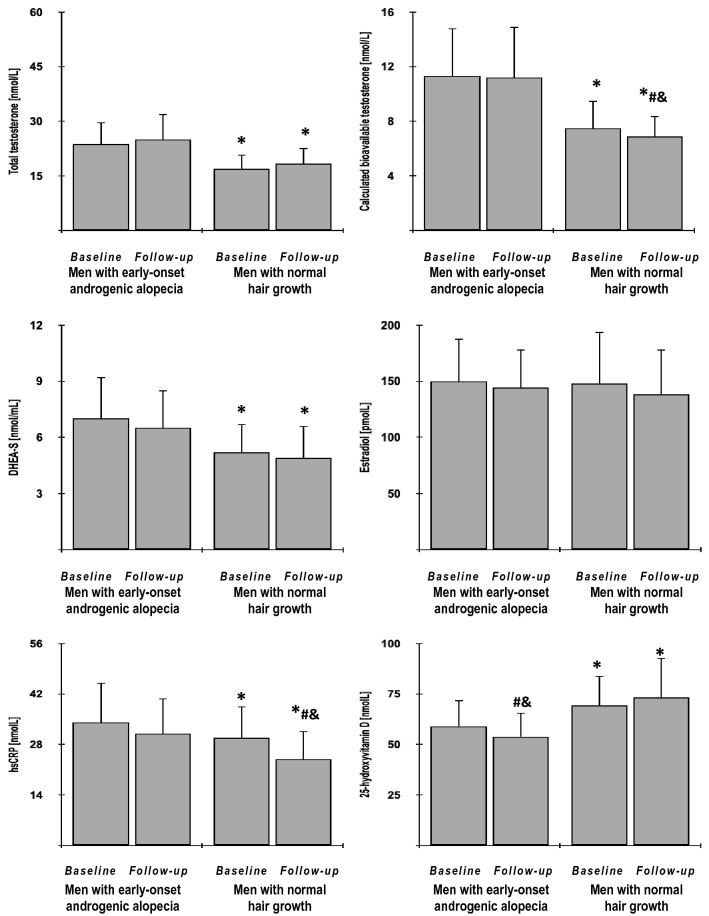
The impact of metformin on hormones, hsCRP and 25-hydroxyvitamin D in the study population. Only data of 135 individuals who completed the study: 65 men with early-onset androgenic alopecia (group A) and 70 men with normal hair growth (group B) were included in the final analyses. Although all values were natural-log transformed, the figure shows the raw data because the mean and SD values of log-transformed data are less relevant. Both groups were compared using Student’s *t*-test for independent samples. Differences between post-treatment (follow-up) and baseline values in each treatment group were identified using Student’s paired t-test. Comparisons of percent changes from baseline after adjustment for baseline values (reflecting the strength of metformin action) were performed using Student's t tests for independent samples. *p* values corrected for multiple testing below 0.05 were considered statistically significant. *p < 0.05 vs. men with early-onset androgenic alopecia in the same time point (group A); ^#^*p* < 0.05 vs. baseline value in the same treatment group; ^&^*p* < 0.05 vs. percent changes from baseline after adjustment for baseline values in the second group

In both study groups, metformin reduced BMI index, fat content, waist circumference, glycated hemoglobin, fasting and postchallenge glucose levels, HOMA1-IR, HOMA2-IR and triglycerides, as well as increased HOMA2-%S, QUICKI, Matsuda index and Stumvoll (0–120) index. With the exception of BMI, these effects were, however, stronger in group B than group A. Only in group B, metformin reduced hsCRP and bioavailable testosterone levels and only in group A the drug reduced 25-hydroxyvitamin D levels. The drug produced a neutral effect on fat-free mass index, blood pressure, HOMA2-%β, total cholesterol, HDL cholesterol, LDL cholesterol, total testosterone, DHEA-S, estradiol, and the estimated glomerular filtration rate. At the end of the study, both groups differed in fat content, waist circumference, glycated hemoglobin, fasting and postchallenge glucose, HOMA1-IR, HOMA2-IR, HOMA2-%S, QUICKI, Matsuda index, Stumvoll (0–120) index, triglycerides, total and bioavailable testosterone, DHEA-S, hsCRP and 25-hydroxyvitamin D (Figs. 2, 3, 4 and 5, Supplementary Table 1).

The impact of metformin on glycated hemoglobin, fasting and postchallenge glucose levels, HOMA1-IR, HOMA2-IR, HOMA2-%S, QUICKI, Matsuda index and Stumvoll (0–120) index correlated with baseline hsCRP levels (group A: *r* values between − 0.321 [*p* = 0.0205] and − 0.553 [*p* < 0.0001]; group B: r values between − 0.304 [*p* = 0.0234] and − 0.568 [*p* < 0.0001]) and baseline 25-hydroxyvitamin D levels (group A: *r* values between 0.311 [*p* = 0.0198] and 0.542 [*p* < 0.0001]; group B: *r* values between 0.325 [*p* = 0.0164] and 0.537 [*p* < 0.0001]). Treatment-induced changes in glucose homeostasis markers correlated in group A with treatment-induced decreases in 25-hydroxyvitamin D levels (*r* values between − 0.328 [*p* = 0.0068] and − 0.510 [*p* < 0.0001]), while in group B with a treatment-induced reduction in hsCRP (*r* values between 0.351 [*p* = 0.0084] and 0.582 [*p* < 0.0001]). Moreover, there were correlations between the effect of metformin treatment on hsCRP and decreases in fat content (group A: *r* = 0.404 [*p* = 0.0006]; group B: *r* = 0.429 [*p* = 0.0004]) and in waist circumference (group A: *r* = 0.341 [*p* = 0.0012]; group B: *r* = 0.385 [*p* = 0.0008]).

## Discussion

The current study shows for the first time that the impact of metformin on glucose homeostasis markers was less pronounced in men with early-onset androgenic alopecia than in their peers with normal growth hair. This finding suggests that individuals with IGT who are at high risk for diabetes may benefit to a lesser degree from metformin treatment if they have coexisting early-onset male pattern hair loss. The results of the study seem clinically relevant because subjects with early-onset androgenic alopecia can be easily selected from the general population based on anamnesis and elementary clinical examination, as well as because they show that early-onset androgenic alopecia cannot be regarded as only a cosmetic defect but can be a real danger to health.

The strength of the current study are strict inclusion and exclusion criteria, allowing us to obtain two homogenous groups of patients. This minimized the possibility that the obtained results resulted from the impact of comorbidities or concomitant therapies. They cannot also be attributed to baseline differences in metabolic characteristics of participants. Because of the selection procedure, duration of IGT, fasting and post-glucose load plasma glucose, glycated hemoglobin, plasma lipids and all indices of insulin sensitivity did not differ between both study populations. Similarities in the inclusion criteria (subjects at high risk for type 2 diabetes who were treated with 1.7 g metformin daily) allow us to relate our findings to the results of the Diabetes Prevention Program, the largest and longest clinical trial of the prevention of diabetes conducted to date [30]. Therefore, the obtained results suggest that the risk of progression to diabetes may be greater in individuals with early-onset androgenic alopecia than in the general population of metformin-treated men.

Compared with the control group, individual with early-onset hair loss were characterized by higher levels of hsCRP and lower concentrations of 25-hydroxyvitamin D. Because similar relationships were found for polycystic ovary syndrome [35, 36], the obtained results are further evidence that biochemical profiles of early-onset androgenic alopecia in men and polycystic ovary syndrome in women closely resemble each other. However, more importantly, the current study provides arguments that differences in metabolic effects of metformin may be partially explained by severity of low-grade systemic inflammation and vitamin D status. Different effects of metformin on glucose homeostasis markers were paralleled by dimorphism in metformin action on hsCRP and both these effects correlated with each other. According to the commonly accepted view, overproduction of proinflammatory cytokines by adipose tissue induces insulin resistance and this effect is partially mediated by systemic inflammation [37]. The results of the present study indicate that different effects on systemic inflammation may reflect between-group differences in the impact of metformin on adipose tissue. In line with this assumption, decreases in fat content and waist circumference in response to metformin treatment were more pronounced in subjects with no evidence of hair loss than in individuals with early-onset androgenic alopecia. Moreover, the impact of metformin on hsCRP levels correlated with treatment-induced changes in fat content and waist circumference, as well as with the changes in the investigated markers of glucose homeostasis.

Observational, cross-sectional, and ecological studies report that vitamin D status is inversely correlated with glycemic control in subjects with type 2 diabetes, as well as with the rate of conversion of prediabetes to diabetes [38]. The role of vitamin D status as a factor determining the strength of metformin action in our study is supported by a different effect of metformin on 25-hydroxyvitamin D concentrations in both groups, as well as by correlations between treatment-induced changes in 25-hydroxyvitamin D levels and metabolic effects of metformin in subjects with early-onset androgenic alopecia. Our findings are in line with previous reports suggesting a beneficial effect of cholecalciferol on metformin action. The impact of metformin on glycated hemoglobin in vitamin D-deficient patients with type 2 diabetes was more pronounced if they were treated with exogenous vitamin D [39], while treatment-induced changes in glucose levels and insulin sensitivity in type 2 diabetic rats were stronger if metformin and exogenous vitamin D were administered together [40]. We cannot explain the reasons for a decrease in 25-hydroxyvitamin D levels only in one study group. However, because metformin is postulated to induce malabsorption of vitamin B_12_ [41] and all subjects withdrawn from the study because of adverse gastrointestinal effects had alopecia, it is possible that worsening of vitamin D status resulted from reduced absorption of this vitamin in the gastrointestinal tract and that men with early-onset male-pattern hair loss are more prone to this complication. Vitamin D may modulate metformin-induced activation of adenosine 5ʹ-monophosphate-activated protein kinase, which is a key cellular sensor of energy homeostasis that regulates glucose and lipid metabolism in specialized metabolic tissues [42], as well as the key molecule in metformin antidiabetic mechanism of action [43]. In line with this hypothesis, vitamin D potentiated metformin action on growth of prostatic cell lines and this effect was mediated by the adenosine 5ʹ-monophosphate-activated protein kinase pathway [44].

Despite the selection, both groups differed in androgen levels, which were higher in individuals with early-onset alopecia, as well as partially in androgen response to metformin treatment. The statistically significant effect of metformin was, however, limited to small changes in bioavailable testosterone levels in men with no evidence of hair loss. This term denotes the sum of free testosterone and testosterone bound loosely to albumin, and reflects the biologically active fraction of testosterone [45]. The lack of correlations with the impact on bioavailable testosterone, indicates, however, that dimorphism in metabolic effects of metformin cannot be directly linked to the androgen status of patients. This observation is in contrast with findings that hypogonadal men and men who underwent androgen deprivation therapy are characterized by increased body weight, increased total fat mass, impaired insulin sensitivity, and are more prone to the development of metabolic syndrome and type 2 diabetes, while testosterone replacement therapy improves glycemic control, increases insulin sensitivity, decreases BMI and visceral fat mass, as well as reduces total and LDL cholesterol levels [46, 47]. This discrepancy is most likely caused by population differences. Small changes in testosterone production, not exceeding some threshold limits, may not determine metabolic effects of metformin, but this does not rule out a modulatory effect of low androgen status or of pharmacologic doses of exogenous testosterone. Dimorphism in metabolic effects of metformin cannot be also attributed to estradiol, which is a product of testosterone aromatization [48]. Despite relatively high concentrations, probably reflecting increased fat content, estradiol levels were similar in both study groups and were unaffected by metformin treatment.

Some study limitations should be mentioned. Although the study was powered and the population exceeded the required number of individuals, the small sample size limits the strength of the conclusions that can be drawn. Measurement of insulin sensitivity by the hyperinsulinemic-euglycemic clamp technique was not performed and the study measured only surrogates of outcome, not investigating clinical outcomes. Despite the fact that the study protocol minimized the impact of random diurnal, seasonal and analytical variations in the outcome measures, the occurrence of the regression-toward-the-mean phenomenon cannot be completely excluded. Finally, because the study protocol did not provide a direct experimental or mechanistic link between metformin action and glucose homeostasis, our conclusions are only speculative.

In conclusion, despite similar baseline levels of glucose homeostasis markers and plasma lipids, men with early-onset androgenic alopecia were characterized by higher levels of androgens and hsCRP and lower levels of 25-hydroxyvitamin D. The impact of metformin on glycated hemoglobin, glucose levels, indices of insulin sensitivity/resistance and triglycerides was more pronounced in men with normal hair growth than in subjects with alopecia, which was accompanied by different effects of this agent on hsCRP and 25-hydroxyvitamin D. These findings suggest that men with early-onset androgenic alopecia may gain less benefits from metformin treatment than other men with prediabetes. Because study limitations make drawing definitive conclusions difficult, the obtained results should be confirmed by large-scale observational and intervention studies.

## Supplementary Information

Below is the link to the electronic supplementary material.

Supplementary file1 (DOCX 23 kb)

## Abbreviations

BMI: Body mass index
DHEA-S: Dehydroepiandrosterone-sulfate
FSH: Follicle-stimulating hormone
HDL: High-density lipoproteins
hsCRP: High-sensitivity C-reactive protein
HOMA1-IR: The homeostasis model assessment 1 of insulin resistance index
hsCRP: High sensitivity C-reactive protein
IGT: Impaired glucose tolerance
LDL: Low-density lipoproteins
LH: Luteinizing hormone
QUICKI: The quantitative insulin sensitivity check index
SD: Standard deviation

## Footnotes

1 High-risk patients with IGT should be treated with metformin if the lifestyle modification is not efficient [26]. Therefore, the study protocol required at least 6 months of non-pharmacological interventions. Because the lifestyle modification had not been always implemented right away, the study included subjects diagnosed with IGT 6–12 months before commencement.

## Declarations

### Institutional approval

The study protocol was approved by the Medical University of Silesia institutional review board.
