# Supplementary material for: Impaired metabolic effects of metformin in men with early-onset androgenic alopecia
Source: Pharmacol Rep. 2021 Dec 12;74(1):216–28. doi: 10.1007/s43440-021-00347-8 (PMC8786753; doi:10.1007/s43440-021-00347-8)
Supplement: Supplementary file 1 — Supplementary file1 (DOCX 23 kb) [file 43440_2021_347_MOESM1_ESM.docx]

**Supplementary Table 1.** The impact of metformin on anthropometric measures, blood pressure, glucose homeostasis markers, plasma lipids, hormones, low-grade systemic inflammation, vitamin D status and estimated glomerular filtration rate in the study population

| **Variable** | **Group A**^a^ | **Group B^b^** | **p-value**  **[Group A vs. Group B]** |
| --- | --- | --- | --- |
| **BMI** [kg/m^2^; mean (SD)] |  |  |  |
| *Baseline* | 32.9 (5.3) | 32.4 (5.1) | 0.5774 |
| *Follow-up* | 31.1 (5.0) | 30.7 (4.8) | 0.6361 |
| *p-value [baseline vs. follow-up]* | **0.0485** | **0.0442** | - |
| **Fat-free mass index** [kg/m^2^; mean (SD)] |  |  |  |
| *Baseline* | 19.0 (2.0) | 18.9 (1.9) | 0.7662 |
| *Follow-up* | 18.6 (2.2) | 19.2 (2.3) | 0.1244 |
| *p-value [baseline vs. follow-up]* | 0.2801 | 0.4016 | - |
| **Fat content** [%; mean (SD)] |  |  |  |
| *Baseline* | 42.2 (4.9) | 41.6 (4.3) | 0.4501 |
| *Follow-up* | 40.2 (4.6) | 37.4 (4.0)* | **0.0002** |
| *p-value [baseline vs. follow-up]* | **0.0179** | **<0.0001** | - |
| **Waist circumference** [cm; mean (SD)] |  |  |  |
| *Baseline* | 108 (8) | 107 (7) | 0.4401 |
| *Follow-up* | 105 (8) | 102 (7)* | **0.0217** |
| *p-value [baseline vs. follow-up]* | **0.0344** | **<0.0001** | - |
| **Systolic blood pressure** [mmHg; mean (SD)] |  |  |  |
| *Baseline* | 125 (11) | 124 (10) | 0.5810 |
| *Follow-up* | 124 (12) | 122 (13) | 0.3557 |
| *p-value [baseline vs. follow-up]* | 0.6213 | 0.3094 | - |
| **Diastolic blood pressure** [mmHg; mean (SD)] |  |  |  |
| *Baseline* | 80 (7) | 79 (7) | 0.4082 |
| *Follow-up* | 80 (6) | 78 (7) | 0.0780 |

| **Glycated hemoglobin** [%, mean (SD)] |  |  |  |
| --- | --- | --- | --- |
| *Baseline* | 6.10 (0.18) | 6.12 (0.15) | 0.4832 |
| *Follow-up* | 5.88 (0.21) | 5.69 (0.16)* | **<0.0001** |
| *p-value [baseline vs. follow-up]* | **<0.0001** | **<0.0001** | - |
| **Fasting glucose** [mmol/L; mean (SD)] |  |  |  |
| *Baseline* | 6.16 (0.41) | 6.11 (0.38) | 0.4634 |
| *Follow-up* | 5.82 (0.36) | 5.58 (0.32)* | **0.0001** |
| *p-value [baseline vs. follow-up]* | **<0.0001** | **<0.0001** | - |
| **2-h post-load glucose** [mmol/L; mean (SD)] |  |  |  |
| *Baseline* | 9.56 (0.78) | 9.44 (0.84) | 0.3924 |
| *Follow-up* | 8.98 (0.82) | 8.55 (0.76)* | **0.0019** |
| *p-value [baseline vs. follow-up]* | **0.0001** | **<0.0001** | - |
| **HOMA1-IR** [mean (SD)] |  |  |  |
| *Baseline* | 4.38 (1.21) | 4.26 (1.25) | 0.5724 |
| *Follow-up* | 3.47 (1.05) | 2.93 (0.97)* | **0.0023** |
| *p-value [baseline vs. follow-up]* | **<0.0001** | **<0.0001** | - |
| **HOMA2-IR** [mean (SD)] |  |  |  |
| *Baseline* | 2.16 (0.32) | 2.11 (0.29) | 0.3427 |
| *Follow-up* | 1.79 (0.25) | 1.56 (0.21)* | **<0.0001** |
| *p-value [baseline vs. follow-up]* | **<0.0001** | **<0.0001** | - |
| **HOMA2-%β** [mean (SD)] |  |  |  |
| *Baseline* | 104.4 (7.8) | 104.6 (7.0) | 0.8755 |
| *Follow-up* | 103.0 (6.0) | 102.4 (6.8) | 0.5888 |
| *p-value [baseline vs. follow-up]* | 0.2535 | 0.0614 | - |
| **HOMA2-%S** [mean (SD)] |  |  |  |
| *Baseline* | 46.6 (7.1) | 47.4 (7.5) | 0.5263 |
| *Follow-up* | 55.9 (8.5) | 64.0 (10.2)* | **<0.0001** |
| *p-value [baseline vs. follow-up]* | **<0.0001** | **<0.0001** | - |
| **QUICKI** [mean (SD)] |  |  |  |
| *Baseline* | 0.308 (0.024) | 0.309 (0.030) | 0.8318 |
| *Follow-up* | 0.318 (0.021) | 0.325 (0.020)* | **0.0494** |
| *p-value [baseline vs. follow-up]* | **0.0127** | **0.0003** | - |
| **Matsuda index** [mean (SD)] |  |  |  |
| *Baseline* | 1.87 (0.32) | 1.91 (0.29) | 0.4475 |
| *Follow-up* | 2.53 (0.41) | 3.01 (0.48)* | **<0.0001** |
| *p-value [baseline vs. follow-up]* | **<0.0001** | **<0.0001** | - |
| **Stumvoll index** [mean (SD)] |  |  |  |
| *Baseline* | 0.0257 (0.0058) | 0.0273 (0.0062) | 0.1246 |
| *Follow-up* | 0.0520 (0.0098) | 0.0634 (0.0112)* | **<0.0001** |
| *p-value [baseline vs. follow-up]* | **<0.0001** | **<0.0001** | - |
| **Total cholesterol** [mmol/L; mean (SD)] |  |  |  |
| *Baseline* | 5.25 (1.32) | 5.12 (1.21) | 0.5515 |
| *Follow-up* | 5.14 (1.26) | 4.95 (1.10) | 0.3515 |
| *p-value [baseline vs. follow-up]*  **HDL cholesterol** [mmol/L; mean (SD)] | 0.6278 | 0.3859 | - |
| *Baseline* | 1.15 (0.24) | 1.18 (0.23) | 0.4597 |
| *Follow-up* | 1.17 (0.25) | 1.24 (0.20) | 0.0737 |
| *p-value [baseline vs. follow-up]* | 0.6425 | 0.1018 | - |
| **LDL cholesterol** [mmol/L; mean (SD)] |  |  |  |
| *Baseline* | 3.20 (0.70) | 3.12 (0.60) | 0.4762 |
| *Follow-up* | 3.15 (0.64) | 3.02 (0.72) | 0.2709 |
| *p-value [baseline vs. follow-up]* | 0.6715 | 0.3736 | - |
| **Triglycerides** [mmol/L; mean (SD)] |  |  |  |
| *Baseline* | 1.91 (0.71) | 1.81 (0.56) | 0.3634 |
| *Follow-up* | 1.70 (0.61) | 1.45 (0.49)* | **0.0094** |
| *p-value [baseline vs. follow-up]* | 0.0728 | **0.0001** | - |
| **Total testosterone** [nmol/L; mean (SD)] |  |  |  |
| *Baseline* | 23.8 (6.0) | 17.0 (3.8) | **<0.0001** |
| *Follow-up* | 25.0 (7.0) | 18.3 (4.4) | **<0.0001** |
| *p-value [baseline vs. follow-up]* | 0.2960 | 0.0635 | - |
| **Calculated bioavailable testosterone** [nmol/L; mean (SD)] |  |  |  |
| *Baseline* | 11.3 (3.5) | 7.5 (2.0) | **<0.0001** |
| *Follow-up* | 11.2 (3.7) | 6.9 (1.5)* | **<0.0001** |
| *p-value [baseline vs. follow-up]* | 0.8434 | **0.0047** | - |
| **DHEA-S** [nmol/mL; mean (SD)] |  |  |  |
| *Baseline* | 7.0 (2.2) | 5.2 (1.5) | **<0.0001** |
| *Follow-up* | 6.5 (2.0) | 4.9 (1.7) | **<0.0001** |
| *p-value [baseline vs. follow-up]* | 0.1775 | 0.2703 | - |
| **Estradiol** [pmol/L; mean (SD)] |  |  |  |
| *Baseline* | 150 (38) | 148 (46) | 0.7843 |
| *Follow-up* | 144 (34) | 138 (40) | 0.3512 |
| *p-value [baseline vs. follow-up]* | 0.3446 | 0.1721 | - |
| **hsCRP** [nmol/L; mean (SD)] |  |  |  |
| *Baseline* | 34.2 (10.8) | 29.8 (8.8) | **0.0103** |
| *Follow-up* | 31.0 (9.8) | 23.9 (7.8)* | **<0.0001** |
| *p-value [baseline vs. follow-up]* | 0.0793 | **<0.0001** | - |
| **25-hydroxyvitamin D** [nmol/L; mean (SD)] |  |  |  |
| *Baseline* | 59.0 (12.8) | 69.4 (14.5) | **<0.0001** |
| *Follow-up* | 53.9 (11.8)* | 73.2 (19.5) | **<0.0001** |
| *p-value [baseline vs. follow-up]* | **0.0197** | 0.1625 | - |
| **Estimated glomerular filtration rate** [ml/min/1.73m^2^; mean (SD)] |  |  |  |
| *Baseline* | 110 (20) | 112 (21) | 0.5725 |
| *Follow-up* | 107 (17) | 108 (18) | 0.7410 |
| *p-value [baseline vs. follow-up]* | 0.3586 | 0.2284 | - |

Only data of 135 individuals who completed the study: 65 men with early-onset androgenic alopecia (group A) and 70 men with normal hair growth (group B) were included in the final analyses. Although all values were natural-log transformed, the figure shows the raw data because the mean and SD values of log-transformed data are less relevant. Both groups were compared using Student’s t-test for independent samples. Differences between post-treatment (follow-up) and baseline values in each treatment group were identified using Student’s paired t-test. Comparisons of percent changes from baseline after adjustment for baseline values (reflecting the strength of metformin action) were performed using Student's t tests for independent samples. *P*-values corrected for multiple testing below 0.05 were considered statistically significant.

^a^Men with early-onset androgenic alopecia

^b^Men with normal hair growth

Statistically significant results are marked in bold.

*p<0.05 vs. percent changes from baseline after adjustment for baseline values in the second group
